# Dietary Polyphenol Intake Inversely Associates with Fibrosis Scores in Individuals with MASLD

**DOI:** 10.3390/nu18203308

**Published:** 2026-10-09

**Authors:** Caterina Bonfiglio, Maria Noemy Pastore, Manuela Siani, Rossella Donghia, Martina Musumeci, Fabio Galvano, Gianluigi Giannelli, Federico Salomone

**Affiliations:** 1 Unit of Data Science, National Institute of Gastroenterology—IRCCS “Saverio de Bellis”, 70013 Castellana Grotte, Italy; catia.bonfiglio@irccsdebellis.it (C.B.); noemy.pastore@irccsdebellis.it (M.N.P.); manuela.siani@irccsdebellis.it (M.S.); rossella.donghia@irccsdebellis.it (R.D.); 2 Division of Gastroenterology, Ospedale di Taormina, Azienda Sanitaria Provinciale di Messina, 98123 Messina, Italy; martinamusu92@gmail.com; 3 Department BioMeTec, University of Catania, 95123 Catania, Italy; fgalvano@unict.it; 4 Scientific Direction, National Institute of Gastroenterology—IRCCS “Saverio de Bellis”, 70013 Castellana Grotte, Italy; gianluigi.giannelli@irccsdebellis.it; 5 Division of Gastroenterology, Ospedale di Acireale, Azienda Sanitaria Provinciale di Catania, 95124 Catania, Italy

**Keywords:** polyphenols, diet, flavonoids, non flavonoids, anthocyanins, MAF-5, fruit, extra virgin olive oil

## Abstract

**Background & aims**. Plant-based dietary patterns, such as the Mediterranean diet, are considered protective against MASLD. The Mediterranean diet is rich in polyphenols (PP), plant-derived metabolites that exhibit anti-fibrotic effects in experimental models. This study aimed to evaluate the association between dietary polyphenol intake and fibrosis scores in patients with MASLD. **Methods**. The study population comprised two retrospective cohorts, MICOL and NUTRIHEP, from southern Italy. Polyphenol intake was assessed using the EPIC food frequency questionnaire, and the food content of polyphenols was quantified using Phenol-Explorer. Liver fibrosis severity was assessed by MAF-5 and FIB-4. **Results**. In a multivariable regression analysis adjusted for several potential confounders, comparing 381 patients with MAF-5 < 0 vs. 94 with MAF-5 > 1, consumption of total PP above the median (382.0–1356.8 mg/day) was associated with lower odds of high MAF-5 (OR 0.49; 95% CI 0.25–0.94; *p* = 0.031). Regarding PP subclasses, individuals with higher intakes of anthocyanins (15.00–252.00 mg/day) and stilbenes (0.04–0.55 mg/day) had lower odds of MAF-5 > 1 (OR 0.52; 95% CI 0.27–0.99; *p* = 0.045 and OR 0.49; 95% CI 0.27–0.92; *p* = 0.025, respectively). Considering PP sources, total flavonoid intake from fruit (≥60 mg/day) was associated with reduced high MAF-5 odds (OR 0.49; 95% CI: 0.24–0.99; *p* = 0.045), driven by fruit-derived anthocyanins (OR 0.52; 95% CI: 0.28–0.97; *p* = 0.04). A higher intake of PP from extra virgin olive oil was also associated with a 50% reduction in the odds of MAF-5 > 1 (OR 0.50; 95% CI: 0.26–0.96; *p* = 0.037). **Conclusions**. In this exploratory study, a higher dietary polyphenol intake may protect against fibrosis in patients with MASLD.

## 1. Introduction

Metabolic dysfunction-associated steatotic liver disease (MASLD) is currently the leading cause of liver cirrhosis and its complications worldwide [1]. Fibrosis onset and progression in subjects with MASLD arise from the interaction between genetic factors and dietary factors [2]. Among dietary factors promoting MASLD, there is large and solid evidence indicating that the intake of saturated fats, fructose, refined carbohydrates, emulsifiers (such as soy lecithin), and non-sugar sweeteners, i.e., components of ultra-processed foods, induces liver steatosis and fibrosis [3]. On the other side, plant-based dietary patterns, such as the Mediterranean Diet (MedDiet), are considered protective against MASLD [4]. The protective effects of the MedDiet against MASLD, and in general against metabolic syndrome, have been attributed to its high content of vegetables, whole grains, fruits, and oily fish [5].

MedDiet foods contain healthy macronutrients such as low glycemic index carbohydrates and polyunsaturated fatty acids, and micronutrients such as vitamins, minerals, fibers, carotenoids and polyphenols [6]. Polyphenols (PPs) are secondary metabolites produced by plants for protection against UV radiation, microbes, or chemical stresses that occur during life; thousands of polyphenols have been isolated and characterized from hundreds of plant-derived foods [7]. Structurally, PPs are made of phenol units, and according to the number and position of aromatic rings and hydroxyl groups, PPs have been classified into four major groups: flavonoids, lignans, phenolic acids, and stilbenes; usually lignans, phenolic acids, and stilbenes are indicated as non-flavonoid PPs. Health benefits derived from PP intake have been largely demonstrated, especially in non-communicable chronic diseases [8], although no universally accepted daily intake threshold for polyphenols has been established. In the MASLD context, Salomone F et al. were the first to show that in an Israeli cohort, a higher intake of phenolic acids was associated with a lower risk of insulin resistance and liver steatosis [9]. Recently, using data from the National Health and Nutrition Examination Survey, He et al. demonstrated that subjects in the highest tertile of flavonoid consumption had 31–34% lower odds of liver steatosis compared to the lowest tertile intake after adjusting for several parameters [10]. However, studies conducted so far have focused only on liver steatosis and metabolic dysfunction, but no study has investigated the effects of dietary PPs on fibrosis, the most robust predictor of liver-related mortality [11]. Therefore, our study aims to investigate the relationship between the intake of dietary flavonoids and non-flavonoids and the fibrosis score in individuals with MASLD living in a Mediterranean diet region.

## 2. Materials and Methods

### 2.1. Study Population

This cross-sectional study was conducted at the National Institute of Gastroenterology “Saverio de Bellis” in Italy (Castellana Grotte, Apulia). The study population comprised two cohorts, details of which have been published elsewhere [12,13,14]. Briefly, NUTRIHEP (Nutrition and Hepatology) is an ongoing prospective study started in 2005–2006, based on a random sample of the Putignano (Apulian Region, Italy) population aged 18 years and older, approved by the Ethical Committee of the Minister of Health (DDG-CE-792/2014, 14 February 2014). The survey aimed to estimate the prevalence of liver diseases and identify the associated dietary patterns within the population. To date, the study has demonstrated a low prevalence of viral hepatitis and a high prevalence of MASLD. The first follow-up was conducted between April 2015 and May 2019 [15].

The MICOL study (Multicenter Italian Cholelithiasis Study) is an ongoing prospective study established in 1985 based on a random sample of the population of Castellana Grotte aged 30 years or older, approved by the National Institute of Gastroenterology (DDG-CE 782/2013). Participants were followed up in 1992–1993, 2005–2006, and 2017–2020; the last follow-up was discontinued due to COVID-19 restrictions. The main objective of the study was to estimate the prevalence of cholelithiasis and its risk factors, but also to assess the prevalence of other liver diseases, such as hepatitis C and MASLD. In the current study, we considered only the 2017–2020 follow-up [16].

### 2.2. Data Collection

Anthropometric and laboratory measurements, as well as liver ultrasound scans, were performed by the same trained staff in both cohorts. The sociodemographic, nutritional, and physical activity questionnaires used were also the same for both cohorts. Blood pressure was assessed in accordance with international guidelines [17]. Weight and height measurements were taken using SECA instruments (Model 700 and Model 206; 220 cm; SECA, Hamburg, Germany). The assessment of the degree of liver steatosis was performed by ultrasound examination (US) for both studies [18]. The liver US parameters are shown in the Supplementary Materials (Figure S1). Fasting blood samples were drawn in the morning and collected in tubes containing ethylenediaminetetraacetic acid (K-EDTA) anticoagulant. The following biomarkers were evaluated in all studies: HbA1c, glucose, AST, ALT, GGT, total cholesterol, HDL cholesterol, triglycerides, insulin, CRP, and complete blood count. The European Prospective Investigation into Cancer and Nutrition (EPIC) questionnaire was self-administered, with staff available to read it if needed. The EPIC food frequency questionnaire (FFQ) was used to document the usual food intake of participants [19].

### 2.3. Outcome Assessment

MAF5 and FIB-4 were chosen as non-invasive tests (NITs) for the evaluation of liver fibrosis in our study [20]. The MAF-5 score was developed to predict at-risk liver stiffness, defined as liver stiffness measurement (LSM) ≥8.0 kPa. The formula includes variables such as waist circumference, BMI, and their interaction. It is extended by assessing the added value of predefined variables known to be associated with liver fibrosis and easily accessible in clinical practice, such as AST, platelets, and diabetes. In the MAF-5 formula, age is intentionally excluded from the variables to prevent misclassification issues seen in NITs that include age, such as FIB-4; for this reason, it is considered the reference NIT in our study.

Both NITs were calculated according to the original formulas. The MAF5 formula is as follows: MAF-5 = −11.3674 + (WC × 0.0282) − (BMI × 0.1761) + (WC × BMI × 0.0019) + (2.0762 if diabetes) + (ln(AST) × 2.9207) − (Platelets × 0.0059) (20). MAF5 was categorized as low (<0), indeterminate (0–1), or high (>1) risk. In our analysis, the intermediate category was excluded from the outcomes [21]. FIB-4 score = Age (years) × AST (U/L)/platelets (109/L) × √ALT (U/L) [22].

FIB-4 is categorized as low (<1.30), indeterminate (1.30–2.67), or high (>2.67) risk of advanced fibrosis using cut-offs previously shown to be associated with advanced fibrosis and in alignment with clinical guidelines (21). In our analysis, the intermediate category (≥1.30 and ≤2.67) was excluded from the outcomes.

### 2.4. Exposure Variables

PP intake was expressed as mg/day and used as the exposure variable in this study. The amounts of PPs in each food group were calculated by summing all the flavonoids and non-flavonoids present in the foods. The intake of PPs from each food was calculated by multiplying the content of each flavonoid and non-flavonoid by the daily consumption of each food. This information was obtained from the Phenol-Explorer database, available online at: http://phenol-explorer.eu/ (accessed on 5 March 2026) [7]. Additionally, the retention factors for each food have been taken into account, since it has been established that cooking methods (boiling, steaming, etc.) might alter the final content of bioactive compounds such as PPs [23].

The structure-based classification of PPs from principal foods in the MedDiet is shown in Figure S2. Among flavonoids, isoflavonoids were excluded from our analysis because of their limited presence in the study sample. The food groups are: vegetables (tomatoes, peppers, cucumbers, aubergines, courgettes, lettuce, broccoli, spinach, artichokes, celery, cabbage, cauliflower, carrots, turnip greens, swiss chard, chicory), legumes (chickpeas, beans, french beans, broad beans, lentils, peas); fruit (grapes, bananas, kiwi, citrus fruits, apples, pears, peaches, apricots, cherries, melon, plums, strawberries) extra virgin olive oil (EVOO) and coffee.

### 2.5. Confounding Variables

Covariates were chosen based on previous research and clinical and statistical judgment regarding their potential links to MAF5 and FIB-4. When considering high MAF5 as an outcome, we included the following covariates: demographic variables (sex, age, marital status, family income assessment), behavioral variables (daily energy intake, relative Mediterranean score (rMED), smoking, and diastolic blood pressure), and blood values (triglycerides, fasting glucose, HDL, and γGT) in models where flavonoids and non-flavonoids provided exposure measures for total intake, vegetables, legumes, and fruit. In models where flavonoids and non-flavonoids in extra virgin olive oil (EVOO) were the exposure variables and MAF5 outcome, we included the following covariates: demographic variables (gender, age, marital status, family income assessment), behavioral variables (relative Mediterranean score (rMED), diastolic blood pressure (DBP), daily energy intake) and blood test results (fasting glucose, γGT, HDL-C, triglycerides). Energy expenditure serves as a reasonable indicator of work done, as it depends on the intensity and type of activity: it can vary from minimal energy use in sedentary jobs to high expenditure in physically demanding roles. To prevent collinearity issues, variables such as waist, BMI, diabetes, AST, and platelets were omitted.

When considering high FIB-4 as an outcome, variables included in the FIB-4 formula, such as age, AST, ALT, and platelets, were excluded. After evaluating potential collinearity, we included the following covariates in models where the outcome was FIB-4: demographic variables (such as sex, family income assessment, and marital status), behavioral factors (including WHR, smoking, diastolic blood pressure, daily energy intake, and relative Mediterranean score [rMED]), and blood biomarkers (triglycerides, fasting glucose, HDL, and γGT). These covariates were used when assessing flavonoids and non-flavonoids as exposure measures for total intake, vegetables, legumes, and fruit.

### 2.6. Statistical Analysis

Individual characteristics are reported as means and standard deviations (M ± SD) or medians and interquartile ranges for continuous variables, and as frequencies and percentages (%) for the categorical variables. We used the Kruskal–Wallis test to compare two groups for continuous variables and the χ^2^ test to evaluate the differences between categorical variables.

We used a logit model to estimate odds ratios (ORs) and 95% confidence intervals (CIs) for both the Fib4 index (<1.30 vs. >2.67) and MAF5 (<0 vs. >1), excluding subjects who fell into the intermediate category as it was not clearly defined as an outcome variable from a clinical point of view.

We also considered the intake of PP, flavonoids, and non-flavonoids (categorized into medians) as exposure variables.

PP, Flavonoids and non-flavonoids were used both as total intake and as contained intake in the following categories: vegetables, legumes, fruits, coffee, and extra virgin olive oil. Since there are no standard thresholds for PPs, flavonoids, and non-flavonoids in the literature, medians were used to determine the cut-offs for exposure variables. Initially, confounding variables were selected from the existing literature. Subsequently, the minimum absolute reduction and selection (LASSO) was employed to decrease the number of candidate predictors and identify those most useful for model construction [24], selecting variables to be included in the model as confounders. In addition, for all models, the variance inflation factor (VIF) was used to assess multicollinearity, and confounders with VIF > 5 were discarded [25,26,27].

A multivariate logistic regression analysis was performed to verify the adjusted association between polyphenol, flavonoid, and non-flavonoid intake and Fib4 and MAF5, correcting for potential confounding factors, including relevant variables that differed between groups, as explained in detail in the section on confounders. All logistic regression analyses were conducted. The estimated coefficients were transformed into odds ratios (OR). The two-tailed probability level was set at 0.05 to test the null hypothesis of non-association. The analyses were conducted with StataCorp 2025 Stata Statistical Software: Release 19 (College Station, TX., USA: StataCorp LLC.).

## 3. Results

### 3.1. Features of the Study Population

**Figure 1:**
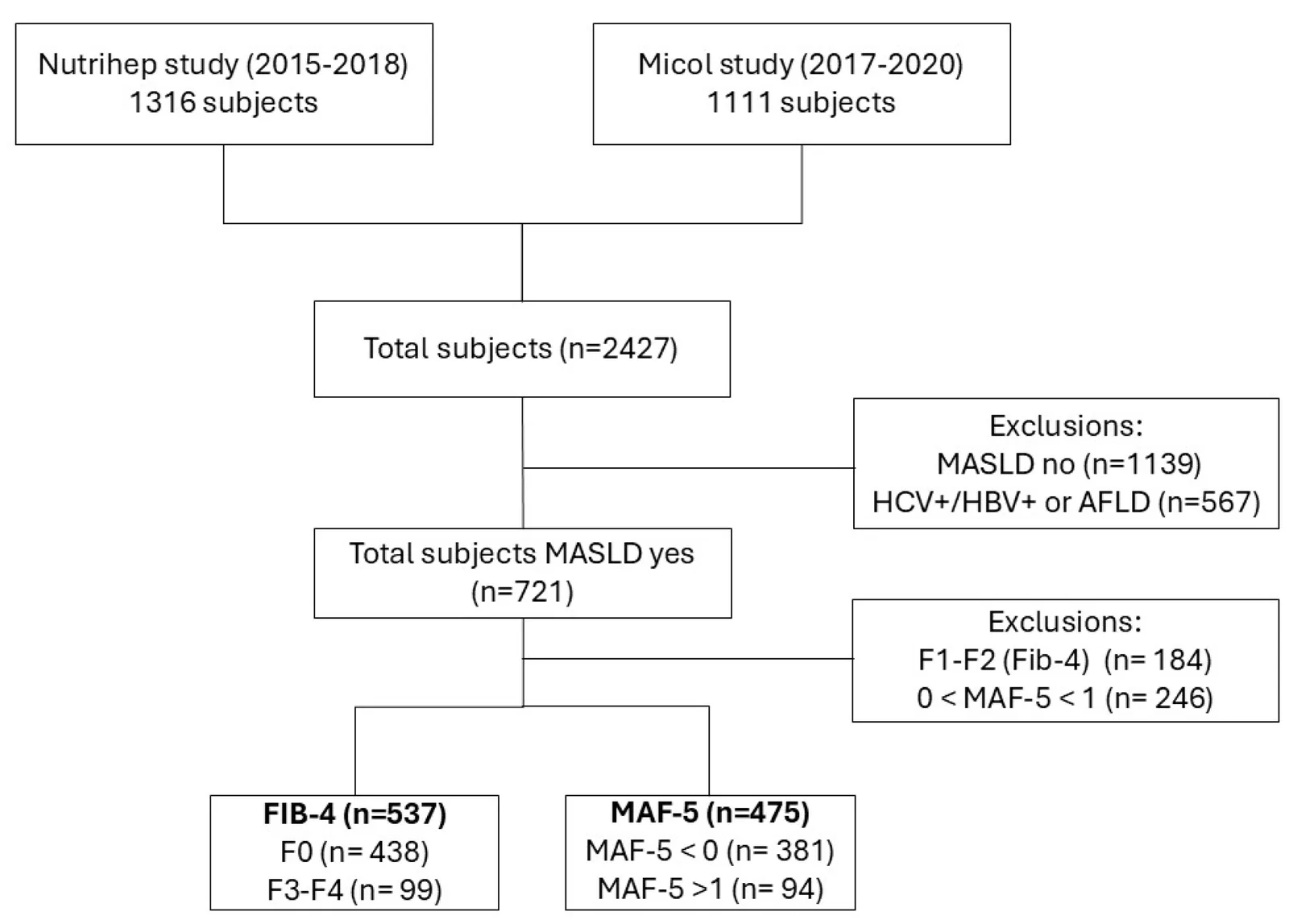
Flow diagram of study participants.

The study population is composed by 2427 subjects, 1316 from the NutriHep cohort e 1111 from the MiCol cohort, as shown in the study flowchart (Figure 1). Among the 2427 subjects, 139 had no US evidence of steatosis and were thus excluded from the analysis. Similarly, 567 subjects were excluded because of the presence of chronic viral hepatitis or alcohol-associated liver disease. Thus, the total number of individuals with MASLD was 721. To better dissect the effect of polyphenol intake on the risk of clinically significant fibrosis, we decided a priori to exclude subjects belonging to the intermediate category of MAF-5 (0–1) or the indeterminate category of FIB-4 (1.30–2.67). Thus, for MAF-5, the number of subjects that underwent the analysis was 475, whereas for FIB-4, the analyses considered 537 individuals (Figure 1).

The anthropometric, clinical, demographic, and lifestyle features of people with MAF-5 < 0 (n = 381) vs. MAF-5 > 1 (n = 94) are summarized in Table 1. As expected, the MAF-5 > 1 group was characterized by a higher prevalence of hypertension (35.78% vs. 59.21%), type 2 diabetes (5.25% vs. 39.36%), and dyslipidemia (15.34% vs. 30.26%), and overall, by a worse cardiometabolic profile, including a higher BMI (28.53 kg/m^2^ vs. 34.97 kg/m^2^), a higher waist-to-hip ratio (0.89 vs. 0.97), higher triglycerides (109.59 mg/dL vs. 128.62 mg/dL) and higher HOMA-IR (2.16 vs. 3.78). In the MAF-5 > 1 group, there was a higher prevalence of males (38.06% vs. 3.83%). Interestingly, the rMED score did not differ between the groups (%), similarly to other lifestyle and demographic factors (Table 1). The clinical and lifestyle features of subjects with FIB-4 < 1.30 vs. FIB-4 > 2.67 are presented in Table S1.

### 3.2. Associations of Dietary Polyphenols with MAF-5 > 1

A multivariate analysis, adjusted for several clinical and lifestyle factors, was performed, and the associations between a MAF-5 > 1 and dietary intake of total polyphenols, total flavonoids, single classes of flavonoids, and single classes of non-flavonoids are presented in Table 2. Firstly, participants whose median total PP intake ranged from 382.0 to 1356.8 showed lower odds of having a MAF-5 > 1 compared to those with an intake between 70.26 and 381.99 mg/day (OR 0.487; 95% CI 0.253–0.938; *p* = 0.031). Similarly, a median intake of total flavonoids from 183.77 to 592.12, versus 16.80 to 183.76, is likely linked to a reduced risk of MAF-5 > 1 (OR 0.515; 95% CI 0.269–0.985; *p* = 0.045). When examining the five classes of flavonoids separately (excluding isoflavones), the intake of anthocyanins was found to have an inverse relationship with high non-invasive fibrosis scores. Consuming a median range of 15.0 to 252.0 mg/day of anthocyanins may lower the odds of a high MAF-5 score compared to intakes of 0–14.99 mg/day (OR 0.521; 95% CI 0.279–0.973; *p* = 0.041).

A probable trend towards a protective effect of the flavonol class was observed (OR 0.593; 95% CI 0.31–1.136; *p* = 0.115). Among the three non-flavonoid classes (phenolic acids, lignans, and stilbenes), only stilbenes were associated with higher non-invasive fibrosis scores. Individuals consuming between 0.039 and 0.55 mg/day might have lower odds of elevated MAF-5 levels compared to those consuming 0 to 0.038 mg/day (OR 0.493; 95% CI 0.265–0.915; *p* = 0.025). The analysis also showed an inverse association with non-flavonoids when comparing participants with a FIB-4 < 1.30 with those with a FIB-4 > 2.67 (OR 0.467; 95% CI 0.231–0.944; *p* = 0.034).

### 3.3. Associations Between Specific Dietary Sources of PP and MAF-5 > 1

A detailed analysis of the food sources of total and single classes of flavonoids and non-flavonoids was also performed. First, we assessed the role of fruit-derived flavonoids in the MAF-5 score (Table 3). Total flavonoid intake from fruits (≥60 mg/day) significantly reduced the odds of a high MAF-5 score (OR 0.486; 95% CI 0.239–0.985; *p* = 0.045). Among the specific flavonoid classes, anthocyanins demonstrated the most substantial benefit (OR 0.521; 95% CI 0.279–0.973; *p* = 0.041). Other subclasses, such as flavanols (*p* = 0.8), flavonols (*p* = 0.6), and flavanones (*p* = 0.6), did not reach statistical significance in the adjusted model.

Analysis of the polyphenols present in extra virgin olive oil (EVOO) has shown that the non-flavonoid components of this food are inversely associated with the risk linked to high scores for non-invasive fibrosis. Each polyphenolic compound analyzed from EVOO showed a significant inverse association with an MAF-5 value > 1. Individuals with an intake above the median for total flavonoids (0.193–2.30) and for total non-flavonoids (7.5–88.87) were less likely to have a MAF-5 score > 1 (OR 0.503; 95% CI 0.263–0.959; *p* = 0.037) respectively (Table 4). Consistent with this, tyrosol (7.48–81.43) and lignans (0.037–0.436) from EVOO had a pooled OR of 0.503 (Table 4). These results were confirmed by the FIB-4 analysis (Supplementary Table S3). Intake above the median for all classes of phenolic compounds in EVOO (flavonoids, non-flavonoids, tyrosols and phenolic acids) was consistently associated with a 50% reduction in the likelihood of having a MAF-5 score > 1 (OR = 0.503; 95% CI: 0.263–0.959; *p* = 0.037). The lignans present in extra virgin olive oil showed an inverse association, with an OR of 0.355 (95% CI: 0.138–0.911; *p* = 0.031).

Finally, non-flavonoids from plant-based sources were particularly influential in the FIB-4 score. As shown in Supplementary Table S4, the intake of vegetable-derived non-flavonoids ≥ 17.0 mg/day was associated with a 71.6% reduction in the odds of high FIB-4 scores (OR 0.284; 95% CI 0.084–0.956; *p* = 0.042). Legume consumption consistently showed an inverse association across multiple phenolic subclasses (Supplementary Table S5).

High intake of non-flavonoids from legumes (≥0.847 mg/day) resulted in an OR of 0.360 (95% CI: 0.147–0.880; *p* = 0.025).

A closer examination of the data reveals that phenolic acids in pulses (≥0.841 mg/day) and stilbenes in pulses (≥0.006 mg/day) both have a statistically significant inverse relationship, with ORs of 0.418 (*p* = 0.047) and 0.328 (*p* = 0.031) respectively. Additionally, non-flavonoid compounds in coffee (≥128.85 mg/day) are associated with a notable reduction in the odds of elevated FIB-4 levels (OR = 0.477; 95% CI: 0.231–0.983; *p* = 0.045), as detailed in Supplementary Table S6. The models that were not statistically significant are shown in Table 5 and Table S7.

Tables S8 and S9 in the supplementary section show that FIb4 and MAF5 were modeled as both continuous variables and divided into three categories. These models used multinomial logit methods to analyze total polyphenols, flavonoids, non-flavonoids, and non-flavonoids in extra virgin olive oil. The sensitivity analyses only partly aligned with the main findings, as some associations that were previously significant no longer remained significant when considering the full outcome categories and non-invasive continuous scores.

## 4. Discussion

This is the first study to evaluate the association between the intake of polyphenolic compounds and the risk of clinically significant fibrosis, as assessed by NITs, in a Mediterranean cohort of subjects with MASLD.

Our findings demonstrate that a higher intake of dietary PPs is significantly associated with lower odds of having a high MAF-5 score or a high FIB-4.

The hepatoprotective effects of PP remained strong even after adjusting for established cardiometabolic factors and adherence to the MedDiet, suggesting that PP has independent hepatoprotective effects from other nutrients that characterize the MedDiet. Our results provide epidemiological evidence to a large amount of data obtained in in vitro and in vivo models of MASLD showing the chemopreventive effects of PP against liver fibrosis [28].

The activity of PPs against MASLD-induced fibrogenesis may depend on pleiotropic effects, including their actions on glucose and lipid homeostasis, insulin sensitivity, inflammation, and oxidative stress, which overall prevent the progression from simple steatosis to steatohepatitis and fibrosis [29]. PP exhibits strong antioxidant properties that protect hepatocytes from lipotoxic damage by directly neutralizing reactive oxygen and nitrogen species or by inducing scavenging enzymes [30]. They modulate key signaling pathways in the activation of hepatic stellate cells, including TGF-β/Smad, AMPK/mTOR, Wnt/β-catenin, NF-κB, PI3K/AKT/mTOR, and Hedgehog [31]. Additionally, due to the importance of the gut-liver axis in MASLD [32], PP contributes to the maintenance of gut microbiota homeostasis, indirectly influencing liver health [33].

Among fruit-derived flavonoids, the intake of anthocyanins showed the strongest inverse association with a high MAF-5 score. These water-soluble pigments, found in berries, plums, red oranges, and grapes, display significant chemopreventive effects against several diseases [34]. In the context of obesity- and metabolic dysfunction-associated diseases, there is evidence both in experimental models and at the clinical level. In a mouse model of high-fat diet-induced obesity, drinking red orange juice exerts hepatoprotective effects through antioxidant activity [35]. A systematic review with a meta-analysis showed that dietary anthocyanins improve metabolic syndrome parameters, making them a promising dietary adjunct for managing metabolic dysfunction [36]. Additionally, anthocyanins exert beneficial effects on gut microbiota, which could contribute to their effectiveness in counteracting metabolically driven fibrosis [32].

Our study also highlights the inverse association of EVOO-derived phenolic compounds. EVOO is a hallmark of the MedDiet, and its phenolic fraction, specifically hydroxytyrosol, tyrosol, oleuropein, and oleocantal, has been widely studied for its beneficial effects in metabolic syndrome. A randomized controlled trial evaluating the impact of olive oil-derived PP in dysmetabolic subjects revealed improvement in cardiometabolic metabolic markers [37]. Research by Gabbia et al. indicated that Tyr and HTyr downregulated markers of HSC activation and reduced oxidative stress and reactive oxygen species (ROS) production [38]. Oleocantal also shows an antifibrotic effect through reduced oxidative stress and inflammation, involving miRNAs that decrease HSC activation and liver fibrosis [39]. Moreover, EVOO-derived PP favorably impacts gut microbiota [40].

One further finding to be underlined concerns the association between a high FIB-4 and a low intake of non-flavonoids derived from vegetables and legumes. Our results show that consuming one standard portion of vegetables per day provides enough PP to help protect against clinically significant liver fibrosis, as assessed by FIB-4 > 2.67. Legumes, rich in fiber and high-quality protein, are a good source of PP [41]. While flavonoids are sensitive to soaking and cooking processes, phenolic acids, particularly hydroxybenzoic acids, can break down during processing [42]. Thus, consuming at least two portions of legumes weekly (approximately 50 g each) can help reduce the likelihood of fibrosis in individuals with MASLD [43]. Plant-derived foods enhance liver health by providing PP and dietary fiber, which promote a healthy gut microbiome and strengthen the gut barrier, thereby decreasing systemic endotoxemia, a key factor in hepatic inflammation [44].

Additionally, although coffee is not specific to the MedDiet, our data confirmed the hepatoprotective effects of coffee-derived non-flavonoids, which is consistent with extensive epidemiological studies that link coffee consumption to a reduced risk of metabolic syndrome and MASLD [45]. A huge number of studies in experimental models indicate that most of the hepatoprotective effects should be attributed to the polyphenolic fraction of coffee rather than to caffeine [46]. In mouse models of MASLD, decaffeinated coffee prevents MASLD by several mechanisms, including modulation of gut microbiota [47], restoration of liver non-coding RNA [48], promotion of chaperones and antioxidant proteins in the liver [49], and induction of nuclear factor erythroid 2-related factor 2 signaling pathway [50].

Our study has strengths, including the use of two NITs, the MAF-5 score, and FIB-4, which provide a comprehensive risk assessment. The study population was composed of two cohorts because this approach led us to hypothesize that the variability among participants could produce more robust results. The two cohorts came from a small town in Southern Italy that strongly adheres to the Mediterranean diet, which we evaluated using the rMed score [51,52] and the EPIC food frequency questionnaire. The EPIC questionnaire allows specific PP subclass assessment, but despite dietitian revision, it is self-reported and may introduce bias. Furthermore, the measurement of PP content was performed using Phenol Explorer, the most comprehensive PP database, by carefully selecting the same food items present in the EPIC questionnaire. It is worth noting that both tools are international, leading to a small discrepancy with local foods. We observed differences in the associations between MAF-5 and FIB-4 and the various PP subclasses. While the FIB-4 index is heavily influenced by age, the MAF-5 score is a new age-independent, anthropometric-based score closely tied to metabolic dysfunction that is characteristic of MASLD [21], and differences may depend on this. The fact that MAF-5 reflects the metabolic dysfunction while FIB-4 mainly indicates the presence of cirrhosis with portal hypertension (it includes platelets) explain the inconsistence of association for certain PPs.

An additional limitation of this study is that physical activity was not assessed; therefore, its potential confounding effect could not be accounted for in the analyses. Future studies should incorporate validated measures of physical activity to enable appropriate adjustment for this potential confounder and to better elucidate the independent associations between the variables investigated [53].

The inherent limitations of non-invasive scores, especially the FIB-4 score’s strong reliance on age, are notable; since age is directly included in the formula, the score increases with age, which can lead to many false positives among older individuals and possible underestimation in younger ones [54].

## 5. Conclusions

In summary, our exploratory research suggests that a higher intake of dietary polyphenols (PPs) may be associated with a lower risk of significant liver fibrosis in a cohort from Southern Italy. These findings are exploratory, as the study is observational and limited to Southern Italy. They suggest a protective association between polyphenols and higher levels of MAF5 and FIB4, but these associations need to be confirmed by further targeted research. The findings should be validated in other populations. Although the cross-sectional design, use of non-invasive scores, and self-reported dietary data may introduce methodological biases, the study appears to confirm the protective role of specific polyphenols found in the Mediterranean diet. It also highlights the importance of personalized nutritional counseling for the management of patients with MASLD at risk of advanced liver disease.

## Figures and Tables

**Table 1:** Anthropometric, clinical, demographic and lifestyle features of people with MAF-5 < 0 (n = 381) vs. MAF-5 > 1 (n = 94).

Parameter ^a^	MAF5 < 0	MAF5 >1	*p*-Value ^b^
N (%)	381 (70.94)	94 (17.50)	
**Anthropometric and clinical parameters**			
SBP (mmHg)	122.60 (14.16)	132.77 (14.08)	<0.001
DBP (mmHg)	78.49 (7.73)	81.33 (7.83)	0.002
Weight (kg)	74.56 (11.79)	93.82 (19.88)	<0.001
BMI (kg/m^2^)	28.53 (4.25)	34.97 (6.65)	<0.001
Waist (cm)	91.82 (9.15)	110.12 (13.03)	<0.001
Hip (cm)	103.67 (8.85)	114.15 (13.15)	<0.001
WHR	0.89 (0.08)	0.97 (0.08)	<0.001
Diabetes (%)			
No	361 (94.75)	57 (60.64)	<0.001
Yes	20 (5.25)	37 (39.36)	
Hypertension (%)			
No	201 (64.22)	31 (40.79)	<0.001
Yes	112 (35.78)	45 (59.21)	
Dyslipidemia			
No	265 (84.66)	53 (69.74)	0.003
Yes	48 (15.34)	23 (30.26)	
**Blood Test**			
ALT (U/L)	23.01 (11.56)	41.37 (50.90)	<0.001
AST (U/L)	21.87 (9.99)	36.18 (39.32)	<0.001
γGT (U/L)	19.26 (17.17)	34.39 (46.15)	<0.001
TC (mg/dL)	196.54 (39.00)	179.37 (43.53)	<0.001
HDL-C (mg/dL)	49.89 (12.57)	43.45 (11.35)	<0.001
TG (mg/dL)	109.59 (73.22)	128.62 (70.47)	0.024
Glucose (mg/dL)	96.63 (18.74)	113.44 (37.80)	<0.001
Insulin (UI/mL)	8.54 (5.68)	13.19 (7.88)	<0.001
HbA1c (mmol/mol)	37.87 (6.45)	41.91 (10.30)	<0.001
HOMA	2.16 (2.15)	3.78 (2.83)	<0.001
*C*-reactive protein (mg/dL)	0.29 (0.60)	0.42 (0.48)	0.070
RBC (10^6^/uL)	4.98 (0.72)	5.00 (0.61)	0.87
WBC (10^3^/uL)	6.36 (3.58)	6.11 (1.79)	0.52
PLT (10^3^/uL)	245.24 (77.96)	208.98 (65.08)	<0.001
**Demographic and lifestyle characteristics**			
Age (years)	56.54 (12.84)	60.03 (13.79)	0.021
Gender (%)			
Female	236 (61.94)	34 (36.17)	<0.001
Male	145 (38.06)	60 (63.83)	
rMED	8.22 (2.41)	7.94 (2.49)	0.34
rMED Score (%)			
Low	93 (24.47)	26 (28.89)	0.40
Moderate	225 (59.21)	54 (60.00)	
High	62 (16.32)	10 (11.11)	
Energy intake (day)	1932.19 (698.86)	1934.68 (768.00)	0.98
Alcohol intake (g/day)	7.87 (13.02)	7.46 (11.65)	0.81
Smoker (%)			
Never/Former	336 (88.19)	84 (89.36)	0.75
Current	45 (11.81)	10 (10.64)	
Marital Status (%)			
Single	38 (9.97)	9 (9.57)	0.22
Married or living together	315 (82.68)	77 (81.91)	
Separated or divorced	10 (2.62)	0 (0.00)	
Widow/er	18 (4.72)	8 (8.51)	
Education (%)			
Primary school	88 (24.93)	25 (30.12)	0.44
Secondary school	118 (33.43)	31 (37.35)	
High school	112 (31.73)	22 (26.51)	
Graduated	35 (9.92)	5 (6.02)	
Family income assessment (%)			
Absolutely insufficient	24 (6.98)	6 (6.38)	0.75
Barely sufficient	61 (17.73)	19 (20.21)	
Sufficient	214 (62.21)	53 (56.38)	
More than sufficient	36 (10.47)	14 (14.89)	
Good, comfortable life	9 (2.62)	2 (2.13)	
Work (%)			
Unemployed & pensioners	103 (27.03)	37 (39.36)	0.09
Managers & professionals	27 (7.09)	3 (3.19)	
Craft, agricultural and sales workers	130 (34.12)	33 (35.11)	
Housewife	52 (13.65)	8 (8.51)	
Elementary occupations	69 (18.11)	13 (13.83)	

^a^ As means and Standard Deviations; ^b^ Kruskal–Wallis test to compare two groups for continuous variables and the χ^2^ test for categorical variables. Legend: SBP: Systolic Blood Pressure; DBP: diastolic blood pressure; BMI: Body Mass Index; WHR: waist-to-hip ratio; ALT: Alanine Aminotransferase; AST: Aspartate Aminotransferase; ɣGT: Gamma Glutamyl Transferase; TC: total cholesterol; HDL-C: High-Density Lipoprotein Cholesterol; TG: triglycerides; HbA1c: Glycosylated Hemoglobin; HOMA: Homeostasis Model Assessment; RBC: Red Blood Cells; WBC: White Blood Cells; PLT: platelets; rMED: Relative Mediterranean Diet.

**Table 2:** Association between polyphenol intake and scores of fibrosis.

Score	MAF5 >1		
	OR	*p*-Value	95% Cl
Median Total Polyphenols (mg/day)			
70.26–381.99	1.000		
382.00–1356.82	0.487	0.031	0.253; 0.938
Median Total Flavonoids (mg/day)			
16.80–183.76	1.000		
183.77–592.12	0.515	0.045	0.269; 0.985
Median Anthocyanins (mg/day)			
0–14.99	1.000		
15.00–252.00	0.521	0.041	0.279; 0.973
Median Flavanols (mg/day)			
0.17–50.31	1.000		
50.32–340.60	0.778	0.433	0.416; 1.457
Median Flavanones (mg/day)			
0–27.62	1.000		
27.63–146.58	0.739	0.343	0.395; 1.381
Median Flavones (mg/day)			
0–27.22	1.000		
27.23–381.79	1.207	0.550	0.652; 2.233
Median Flavonols (mg/day)			
0.80–42.06	1.000		
42.07–379.72	0.593	0.115	0.310; 1.136
Median Total Non-Flavonoids (mg/day)			
15.95–185.49	1.000		
185.50–501.84	0.923	0.800	0.498; 1.710
Median Phenolic Acids (mg/day)			
2.22–185.20	1.000		
185.21–433.98	0.887	0.713	0.470; 1.676
Median Lignans (mg/day)			
0.019–0.412	1.000		
0.413–2.015	0.695	0.240	0.379; 1.275
Median Stilbenes (mg/day)			
0–0.038	1.000		
0.039–0.55	0.493	0.025	0.265; 0.915

MAF5 < 0 as referent category. Models adjusted for gender, age, smoking, γGT, triglycerides, HDL-C, fasting glucose, DBP, daily energy intake, rMED, marital status, and family income assessment. Legend: WHR: waist-to-hip ratio; ɣGT: Gamma Glutamyl Transferase; HDL-C: High-Density Lipoprotein Cholesterol; DBP: diastolic blood pressure; daily energy intake, rMED: Relative Mediterranean Diet; OR: odds ratio.

**Table 3:** Association between total flavonoids and specific flavonoid classes from fruit and MAF5.

Score	MAF5 > 1		
	OR	*p*-Value	95% CI
Median total flavonoids from fruit (mg/day)			
1.04–60.00	1.000		
60.01–547.74	0.486	0.045	0.239; 0.985
Median flavanols from fruit (mg/day)			
0.152–3.110	1.000		
3.111–59.78	0.808	0.531	0.415; 1.572
Median flavonols from fruit (mg/day)			
0.119–1.63	1.000		
1.64–15.99	0.601	0.125	0.314; 1.152
Median flavanones from fruit (mg/day)			
0.00–27.60	1.000		
27.61–77.30	0.636	0.186	0.326; 1.243
Median anthocyanins from fruit (mg/day)			
0–14.99	1.000		
15.00–252.00	0.521	0.041	0.279; 0.973

MAF5 < 0 referent category. Model b: MAF5 < 0 as referent category. Model adjusted for gender, age, smoking, γGT, triglycerides, HDL-C, fasting glucose, diastolic blood pressure, daily energy intake, rMED, marital status, and family income assessment. Legend: ɣGT: Gamma Glutamyl Transferase; HDL-C: High-Density Lipoprotein Cholesterol; rMED: Relative Mediterranean Diet; OR: odds ratio. Flavanones from oranges. Flavanols from apricots, bananas, kiwis, apples, pears, peaches, grapes, plums and strawberries. Flavonols from: apricots, apples, pears, grapes, oranges, plums, strawberries. Anthocyanins from: grapes, plums, strawberries, cherry.

**Table 4:** Association between the intake of flavonoids and non-flavonoids from EVOO and MAF-5.

Score		MAF5 > 1	
	OR	*p*-Value	95% CI
Median Flavonoids from EVOO: (mg/day)			
0.012–0.192	1.000		
0.193–2.30	0.503	0.037	0.263; 0.959
Median Non-Flavonoids from EVOO: (mg/day)			
0.472–7.64	1.000		
7.65–88.87	0.503	0.037	0.263; 0.959
Median Tyrosols from EVOO (mg/day)			
0.475–7.47	1.000		
7.48–81.43	0.503	0.037	0.263; 0.959
Median Phenolic Acids from EVOO: (mg/day)			
0.002–0.036	1.000		
0.037–0.436	0.503	0.037	0.263; 0.959
Median Lignans from EVOO: (mg/day)			
0.008–0.135	1.00		
0.136–1.626	0.503	0.037	0.263; 0.959

MAF5 < 0 referent category. Model b: MAF5 < 0 as referent category. Model adjusted for gender, age, γGT, triglycerides, HDL-C, fasting glucose, diastolic blood pressure, daily energy intake, rMED, marital status, and family income assessment. Legend: ɣGT: Gamma Glutamyl Transferase; HDL-C: High-Density Lipoprotein Cholesterol; rMED: Relative Mediterranean Diet; OR: odds ratio.

**Table 5:** Non-statistically significant associations between flavonoids, non-flavonoids (subdivided by median value), and MAF5.

MAF5 > 1			
	OR	*p*-Value	95% CI
Flavonoids from vegetables (mg/day)			
<41.31	1.000		
≥41.31	1.274	0.485	0.646; 2.515
Non-flavonoids from vegetables (mg/day)			
<10.67	1.000		
≥10.67	0.696	0.304	0.348; 1.390
Flavonoids from legumes (mg/day)			
<1.67	1.000		
≥1.67	1.501	0.225	0.779; 2.891
Non-flavonoids from legumes (mg/day)			
<0.847	1.000		
≥0.847	1.370	0.349	0.709; 2.647
Non-flavonoids from fruit (mg/day)			
<43.32	1.000		
≥43.32	0.604	0.163	0.298; 1.225
Non-flavonoids from coffee (mg/day)			
<128.85	1.000		
≥128.85	1.591	0.153	0.841; 3.007

MAF5 < 0 referent category. Models adjusted for gender, age, smoking, γGT, triglycerides, HDL-C, fasting glucose, DBP, daily energy intake, rMED, marital status, and family income assessment. Legend: ɣGT: Gamma Glutamyl Transferase; HDL-C: High-Density Lipoprotein Cholesterol; DBP: diastolic blood pressure; rMED: Relative Mediterranean Diet; OR: odds ratio.

## Data Availability

The original data presented in the study are openly available https://doi.org/10.6084/m9.figshare.33033545.

## Supplementary Materials

The following supporting information can be downloaded at: https://www.mdpi.com/article/10.3390/nu18203308/s1, Figure S1. Liver Ultrasound; Figure S2. Classification of polyphenols and main MedDiet foods that contain them; Table S1. Anthropometric, clinical, demographic, and lifestyle features of people with FIB-4 < 1.30 (n = 438) vs. FIB-4 > 2.67 (n = 99); Table S2. Association between polyphenol intake and scores of fibrosis; Table S3. Association between intake of flavonoids, non-flavonoids and specific phenolic compounds from EVOO and FIB4 > 2.67; Table S4. Association between intake of non-flavonoids from vegetables and FIB4 > 2.67; Table S5. Association between intake of non-flavonoids from legumes and FIB4 > 2.67; Table S6. Association between intake of non-flavonoids from coffee and FIB4 > 2.67; Table S7. Non-statistically significant associations between flavonoids, non-flavonoids (subdivided by median value) and Fib4; Table S8. Association between polyphenol intake and scores of fibrosis Fib 4; Table S9. Association between polyphenol intake and scores of fibrosis MAF 5.

## Abbreviations

EPIC	European Prospective Investigation into Cancer and Nutrition
EVOO	Extra Virgin Olive Oil
MASLD	Metabolic Dysfunction-Associated Liver Disease
MedDiet	Mediterranean Diet
MICOL	Multicenter Italian Cholelithiasis
NUTRIHEP	Nutrition and Hepatology
PP	Polyphenols
