# Supplementary material for: Dietary Polyphenol Intake Inversely Associates with Fibrosis Scores in Individuals with MASLD
Source: Nutrients. 2026 Oct 9;18(20):3308. doi: 10.3390/nu18203308 (PMC13708400; doi:10.3390/nu18203308)
Supplement: Supplementary file 1 [file nutrients-18-03308-s001.zip › nutrients-4581765-supplementary.pdf]

SUPPLEMENTARY MATERIALS

Figure S1. Liver Ultrasound

**Dimensions**	Normal	Increase	Reduced
**Margins**	Regular	Irregulars	
**Ecostructure**	Homogeneous Normoechogenic	Uneven	Hyperechogenic

Evaluation of Hepatic Steatosis

**Contrast between liver parenchyma (P. EPA) and renal parenchyma (P. REN)**	**Deep beam penetration ultrasonic**	**Sharpness of the vascular structures, particularly particularly the veins**
Homogeneous echo level and contrast between P. EPA and P. REN not evident (0)	Hepatic parenchyma clearly visible from the surface to the diaphragm (0)	Vascular structures clearly visible (0)
Slight discrepancy in echogenicity hepatic-renal (1)	Presence of opacity of the deeper parts of the liver or failure to visualize the diaphragm (1)	Loss of echoes of the vascular structures (1)
Wide discrepancy between hepatic and renal (2)	Presence of opacity of the deeper parts of the liver and non-visualisation of the diaphragm (2)	Vascular structures not clearly visible (2)
**Steatosis score**: Steatosis Absent (0); Mild Steatosis (1-2); Moderate Steatosis (3-5); Severe Steatosis (6)		

Examination performed with Esaote MyLab70 XVG device and 5 MHz Convex probe

Figure S2. Classification of polyphenols and main MedDiet foods that contain them

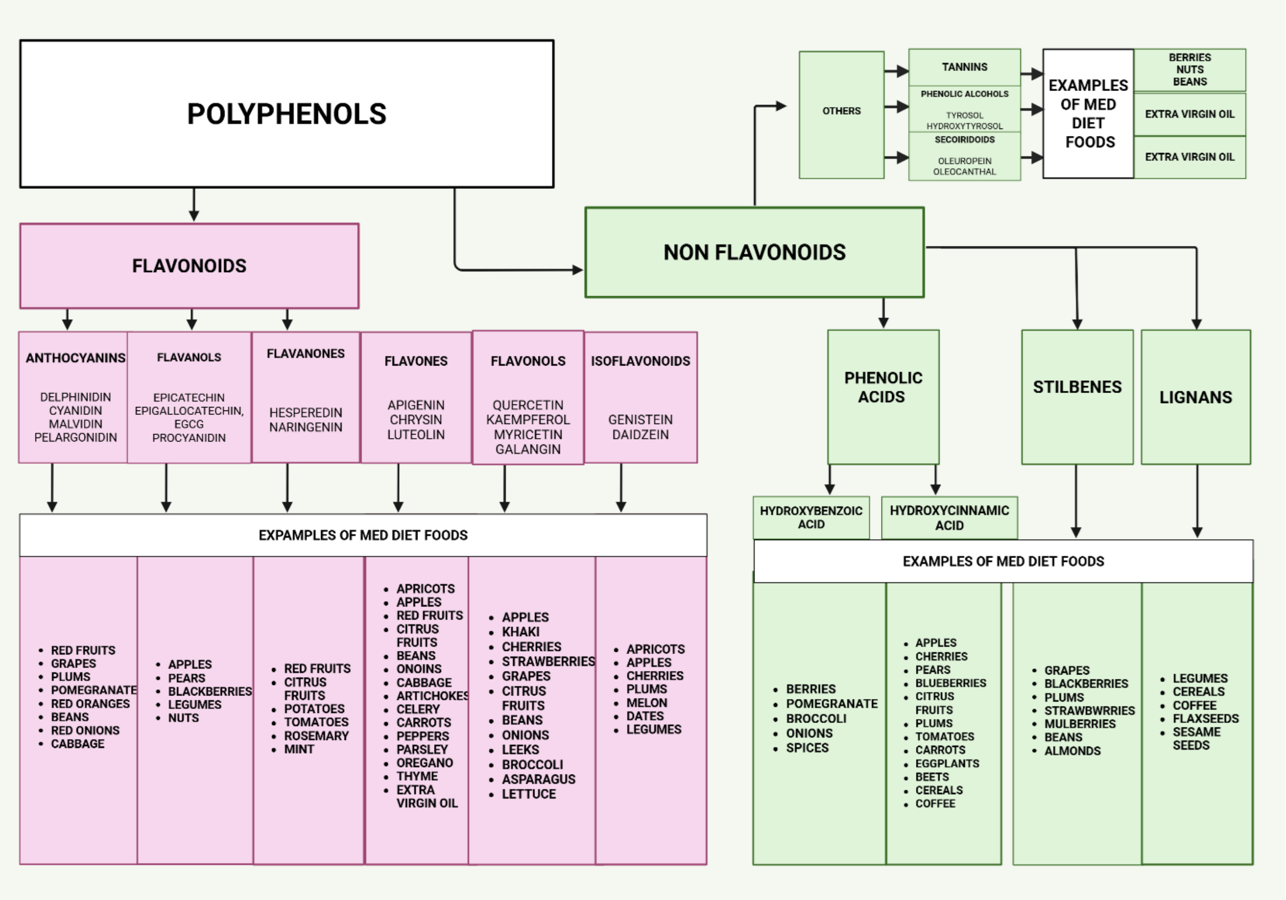

Table S1. Anthropometric, clinical, demographic and lifestyle features of people with FIB-4 < 1.30 (n=438) vs. FIB-4 > 2.67 (n=99)

Parameter [a]	**FIB-4 < 1.30**	**FIB-4 > 2.67**	p-value
N (%)	438 (81.56)	99 (18.43)	
**Anthropometric and clinical parameters**			
SBP (mmHg)	123.00 (13.80)	133.71 (14.46)	<0.001
DBP (mmHg)	79.56 (7.67)	78.04 (7.96)	0.079
Weight (kg)	80.88 (15.46)	72.50 (14.26)	<0.001
BMI (kg/m^2)	30.43 (5.32)	28.63 (5.31)	0.002
Waist (cm)	96.89 (12.42)	95.51 (11.96)	0.38
Hip (cm)	106.56 (10.84)	104.66 (10.12)	0.17
WHR	0.91 (0.09)	0.91 (0.08)	0.86
Diabetes (%)			
No	389 (88.81)	80 (80.81)	0.031
Yes	49 (11.19)	19 (19.19)	
Hypertension (%)			
No	222 (64.16)	33 (35.87)	<0.001
Yes	124 (35.84)	59 (64.13)	
Dyslipidaemia			
No	296 (85.55)	63 (68.48)	<0.001
Yes	50 (14.45)	29 (31.52)	
**Blood test**			
ALT (U/L)	25.07 (11.89)	35.02 (51.39)	<0.001
AST (U/L)	21.24 (5.61)	39.42 (40.30)	<0.001
γGT (U/L)	19.27 (13.70)	37.65 (53.03)	<0.001
TC (mg/dL)	199.05 (38.64)	169.02 (41.99)	<0.001
HDL-C (mg/dL)	48.29 (11.51)	48.26 (15.48)	0.98
TG (mg/dL)	115.01 (69.96)	115.52 (83.17)	0.95
Glucose (mg/dL)	97.96 (19.37)	108.46 (35.64)	<0.001
Insulin (UI/mL)	9.77 (5.86)	9.47 (8.80)	0.68
HbA1c (mmol/mol)	38.62 (7.43)	39.24 (9.31)	0.60
HOMA	2.46 (1.80)	2.81 (3.80)	0.17
C-reactive protein (mg /dL)	0.33 (0.59)	0.23 (0.26)	0.26
RBC (10^6/uL)	5.03 (0.54)	4.78 (1.11)	<0.001
WBC (10^3/uL)	6.28 (1.64)	6.27 (6.52)	0.98
PLT (10^3/uL)	257.91 (64.96)	143.87 (39.99)	<0.001
**Demographic and lifestyle characteristics**			
Age (years)	53.53 (10.56)	73.84 (9.91)	<0.001
Gender (%)			
Female	264 (60.27)	35 (35.35)	<0.001
Male	174 (39.73)	64 (64.65)	
rMED	8.14 (2.46)	8.19 (2.31)	0.86
rMED Score (%)			
Low	117 (26.90)	23 (23.96)	0.77
Moderate	250 (57.47)	59 (61.46)	
High	68 (15.63)	14 (14.58)	
Energy intake (day)	1,950.37 (724.68)	1,853.90 (650.12)	0.23

Alcohol intake (g/day)	8.11 (12.97)	3.56 (8.20)	0.097
Smoker (%)			
Never/Former	382 (87.21)	95 (95.96)	0.013
Current	56 (12.79)	4 (4.04)	
Marital Status (%)			
Single	50 (11.42)	3 (3.03)	<0.001
Married or living together	359 (81.96)	83 (83.84)	
Separated or Divorced	13 (2.97)	0 (0.00)	
Widow/er	16 (3.65)	13 (13.13)	
Education (%)			
Primary school	72 (18.14)	58 (61.05)	<0.001
Secondary school	148 (37.28)	19 (20.00)	
High School	135 (34.01)	15 (15.79)	
Graduated	42 (10.58)	3 (3.16)	
Family income assessment (%)			
Absolutely insufficient	29 (7.13)	4 (5.26)	0.001
Barely sufficient	74 (18.18)	10 (13.16)	
Sufficient	256 (62.90)	43 (56.58)	
More than sufficient	37 (9.09)	19 (25.00)	
Good, comfortable life	11 (2.70)	0 (0.00)	
Work (%)			
Unemployed & Pensioners	89 (20.32)	77 (77.78)	<0.001
Managers & Professionals	33 (7.53)	2 (2.02)	
Craft, Agricultural and Sales Workers	167 (38.13)	14 (14.14)	
Housewife	63 (14.38)	3 (3.03)	
Elementary Occupations	86 (19.63)	3 (3.03)	

[a]As means and Standard Deviations. [b]Percentages calculated for the column.

Legend: SBP: Systolic Blood Pressure; DBP: Diastolic Blood Pressure; BMI: Body Mass Index; WHR: Waist-to-Hip Ratio; ALT: Alanine Aminotransferase; AST: Aspartate Aminotransferase; ɣGT: Gamma Glutamyl Transferase; TC: Total Cholesterol; HDL-C: High-Density Lipoprotein Cholesterol; TG: Triglycerides; HbA1c: Glycosylated Haemoglobin; HOMA: Homeostasis Model Assessment; RBC: Red Blood Cells; WBC: White Blood Cells; PLT: Platelets; rMED: Relative Mediterranean Diet;

Table S2. Association between polyphenol intake and scores of fibrosis

Score	FIB4 >2.67		
	OR	p-value	95% Cl
Median total polyphenols (mg/day)			
70.26 – 381.99	1.000		
382.00 - 1356.82	0.597	0.315	0.219; 1.631
Median total flavonoids (mg/day)			
16.80 - 183.76	1.000		
183.77 – 592.12	1.410	0.470	0.555; 3.585
Median Anthocyanins (mg/day)			
0 - 14.99	1.000		
15.00 - 252.00	1.849	0.135	0.826; 4.136
Median Flavanols (mg/day)			
0.17 - 50.31	1.000		
50.32 - 340.60	1.694	0.218	0.733; 3.916
Median Flavanones (mg/day)			
0 - 27.62	1.000		
27.63 - 146.58	1.674	0.648	0.784; 3.576
Median Flavones (mg/day)			
0 - 27.22	1.000		
27.23 - 381.79	1.111	0.786	0.520; 2.373
Median Flavonols (mg/day)			
0.80 - 42.06	1.000		
42.07 - 379.72	1.375	0.403	0.652; 2.899
Median total non-flavonoids (mg/day)			
15.95 - 185.49	1.000		
185.50 - 501.84	0.467	0.034	0.231; 0.944
Median Phenolic Acids (mg/day)			
2.22 - 185.20	1.000		
185.21 - 433.98	0.453	0.048	0.207; 0.993
Median Lignans (mg/day)			
0.019 - 0.412	1.000		
0.413 - 2.015	1.427	0.310	0.718; 2.834
Median Stilbenes (mg/day)			
0 - 0.038	1.000		
0.039 – 0.55	1.922	0.077	0.933; 3.962

FIB-4<1.30 as referent category. Models adjusted for gender, smoke, WHR, DBP, γGT, Triglycerides, HDL-C, Fasting Glucose, Daily Energy Intake, rMED, Marital Status, Family income assessment.
Legend: WHR: Waist-to-Hip Ratio; ɣGT: Gamma Glutamyl Transferase; HDL-C: High-Density Lipoprotein Cholesterol; DBP: Diastolic Blood Pressure; Daily Energy Intake, rMED: Relative Mediterranean Diet; OR: Odds Ratio

Table S3. Association between intake of flavonoids, non-flavonoids and specific phenolic compounds from EVOO and FIB4>2.67

Score	FIB4>2.67		
	OR	p-value	95% CI

Median flavonoids from EVOO: (mg/day)			
0.012 - 0.192	1.000		
0.193 – 2.30	0.390	0.034	0.163; 0.932
Median non-flavonoid from EVOO: (mg/day)			
0.472 - 7.64	1.000		
7.65 - 88.87	0.390	0.034	0.163; 0.932
Median tyrosol in EVOO (mg/day)			
0.475 - 7.47	1.000		
7.48 - 81.43	0.390	0.034	0.163; 0.932
Median phenolic acids from EVOO: (mg/day)			
0.002 - 0.036	1.000		
0.037 - 0.436	0.390	0.034	0.163; 0.932
Median lignans from EVOO: (mg/day)			
0.008 - 0.135	1.00		
0.136 - 1.626	0.390	0.034	0.163; 0.932

FIB-4<1.30 as referent category. Model adjusted for gender, WHR, Diastolic Blood Pressure, γGT, Triglycerides, HDL-C, Fasting Glucose, rMED, Daily Energy Intake, Marital Status, Family income assessment. Legend: WHR: Waist-to-Hip Ratio; ɣGT: Gamma Glutamyl Transferase; HDL-C: High-Density Lipoprotein Cholesterol; rMED: Relative Mediterranean Diet; OR: Odds Ratio; CI: Confident Interval

Table S4. Association between intake of non-flavonoids from vegetables and FIB4>2.67

Score	FIB4>2.67		
	OR	p-value	95% CI
Median non-flavonoids from vegetables (mg/day)			
0.13 - 17.00	1.000		
17.01 - 140.77	0.284	0.042	0.084; 0.956
Median phenolic acids from vegetables (mg/day)			
0.13 – 16.00	1.00		
16.01 – 139.15	0.283	0.042	0.084; 0.957
Median Lignans from vegetables (mg/day)			
0.00 – 0.06	1.00		
0.07 - 1.61	1.009	0.982	0.445; 2.287

FIB-4<1.30 as referent category. Model adjusted for gender, smoke, WHR, DBP, γGT, Triglycerides, HDL-C, Fasting Glucose, Daily Energy Intake, rMED, Marital Status, Family income assessment. Legend: WHR: Waist-to-Hip Ratio; ɣGT: Gamma Glutamyl Transferase; DBP: Diastolic Blood Pressure, HDL-C: High-Density Lipoprotein Cholesterol; rMED: Relative Mediterranean Diet; OR: Odds Ratio

Table S5. Association between intake of non-flavonoids from legumes and FIB4>2.67

Score	FIB4>2.67		
	OR	p-value	95% CI
Median non-flavonoids from legumes: (mg/day)			
0.00 - 0.847	1.000		
0.848 – 11.31	0.360	0.025	0.147; 0.880
Median phenolic acids from legumes: (mg/day)			
0.00 - 0.841	1.000		
0.842 – 11.22	0.418	0.047	0.177; 0.990
Median stilbenes from legumes: (mg/day)			
0.00 - 0.006	1.00		
0.007 - 0.089	0.328	0.031	0.119; 0.905

FIB-4<1.30 as referent category Model adjusted for gender, smoke, WHR, DBP, γGT, Triglycerides, HDL-C, Fasting Glucose, Daily Energy Intake, rMED, Marital Status, Family income assessment. Legend: WHR: Waist-to-Hip Ratio; ɣGT: Gamma Glutamyl Transferase; HDL-C: High-Density Lipoprotein Cholesterol; DBP: Diastolic Blood Pressure, rMED: Relative Mediterranean Diet; OR: Odds Ratio

Table S6. Association between intake of non-flavonoids from coffee and FIB4>2.67

Score	FIB4>2.67		
	OR	p-value	95% CI
Median non-flavonoids from coffee: (mg/day)			
0.00 - 128.85	1.000		
128.86 - 386.55	0.477	0.045	0.231; 0.983

FIB-4<1.30 as referent category. Model adjusted for gender, smoke, Systolic Blood Pressure γGT, Triglycerides, HDL-C, Fasting Glucose, rMED, Daily Energy Intake, Marital Status, Family income assessment. Legend: WHR: Waist-to-Hip Ratio; ɣGT: Gamma Glutamyl Transferase; HDL-C: High-Density Lipoprotein Cholesterol; rMED: Relative Mediterranean Diet; OR: Odds Ratio

Table S7. Non-statistically significant associations between flavonoids, non-flavonoids (subdivided by median value) and Fib4.

	Fib4 = F3F4		
	OR	p-value	95% CI
Flavonoids from vegetables (mg/day)			
< 41.31	1.000		
≥41.31	0.947	0.901	0.400; 2.239
Flavonoids from fruit (mg/day)			
<83.40	1.000		

≥83.40	1.369	0.499	0.551; 3.402
Non-flavonoids from fruit (mg/day)			
<43.32	1.000		
≥43.32	1.977	0.147	0.786; 4.969
Flavonoids from legumes (mg/day)			
<1.67	1.000		
≥1.67	0.608	0.280	0.247; 1.500

Fib4<1.30 referent category. Model adjusted for gender, smoke, WHR, DBP, γGT, Triglycerides, HDL-C, Fasting Glucose, Daily Energy Intake, rMED, Marital Status, Family income assessment. Legend: WHR: Waist-to-Hip Ratio; γGT: Gamma Glutamyl Transferase; DBP: Diastolic Blood Pressure, HDL-C: High-Density Lipoprotein Cholesterol; rMED: Relative Mediterranean Diet; OR: Odds Ratio

Table S8. Association between polyphenol intake and scores of fibrosis Fib 4

	**1.30 ≤FIB-4≤1.30**			**FIB4 >2.67**			**Trend (continuous value)**		
	RRR	*p-value*	95% Cl	RRR	*p-value*	95% Cl	β	*p-value*	95% Cl
Median total polyphenols (mg/day)							0.000		
70.26 – 381.99	1.000			1.000			-0.078	0.102	-0.172; 0.016
382.00 - 1356.82	0.991	0.964	0.679; 1.447	0.992	0.988	0.371; 2.655			
Median total flavonoids (mg/day)									
16.80 - 183.76	1.000			1.000			0.000		
183.77 – 592.12	1.334	0.139	0.910; 1.953	1.823	0.245	0.663; 5.013	-0.010	0.844	-0.106; 0.087
Median total non-flavonoids (mg/day)									
15.95 - 185.49	1.000			1.000			0.000		
185.50 - 501.84	0.782	0.187	0.542; 1.127	0.698	0.465	0.265; 1.834	-0.086	0.055	-0.174; 0.002
Median non-Flavonoids from EVOO: (mg/day)									
0.472 – 7.64	1.000			1.000			0.000		
7.65 – 88.87	0.294	0.172	0.051; 1.702	0.563	0.008	0.367; 0.862	-0.087	0.045	-0.171; -0.002

FIB-4 < 1.30 as base outcome. Models adjusted for gender, smoke, WHR, DBP, γGT, Triglycerides, HDL-C, Fasting Glucose, Daily Energy Intake, rMED, Marital Status, Family income assessment. Model for EVOO adjusted for gender, smoke, WHR, DBP, γGT, Triglycerides, HDL-C, Fasting Glucose, Daily Energy Intake, rMED, Marital Status, Family income assessment. Legend: WHR: Waist-to-Hip Ratio; ɣGT: Gamma Glutamyl Transferase; HDL-C: High-Density Lipoprotein Cholesterol; DBP: Diastolic Blood Pressure; Daily Energy Intake; rMED: Relative Mediterranean Diet; RRR: Relative Risk Ratios.

Table S9. Association between polyphenol intake and scores of fibrosis MAF 5

	0≤MAF5≤1			MAF5<1			Trend (continuous value)		
	RRR	*p-value*	95% Cl	RRR	*p-value*	95% Cl	β	*p-value*	95% Cl
Median total polyphenols (mg/day)									
70.26 – 381.99	1.000			1.000			0.000		
382.00 - 1356.82	0.892	0.695	0.505; 1.575	0.799	0.435	0.455; 1.403	-0.119	0.381	-0.386; 0.148
Median total flavonoids (mg/day)									
16.80 - 183.76	1.000			1.000			0.000		
183.77 – 592.12	1.308	0.265	0.816; 2.099	0.597	0.049	0.357; 0.997	-0.215	0.121	-0.488; 0.057
Median total non-flavonoids (mg/day)									
15.95 - 185.49	1.000			1.000			0.000		
185.50 - 501.84	0.838	0.533	0.480; 1.461	0.966	0.901	0.558; 1.671	-0.148	0.244	-0.397; 0.101
Median non-Flavonoids from EVOO: (mg/day)									
0.472 – 7.64	1.000			1.000			0.000		
7.65 – 88.87	0.735	0.333	0.394; 1.371	0.488	0.026	0.260; 0.916	0.031	0.686	-0.118; 0.180

MAF5<0 as base outcome. Models adjusted for gender, age, smoke, γGT, Triglycerides, HDL-C, Fasting Glucose, DBP, Daily Energy Intake, rMED. Marital Status, Family income assessment. Model for EVOO adjusted for gender, age, γGT, Triglycerides, HDL-C, Fasting Glucose, Diastolic Blood Pressure, Daily Energy Intake, rMED. Marital Status, Family income assessment. Legend: WHR: Waist-to-Hip Ratio; ɣGT: Gamma Glutamyl Transferase; HDL-C: High-Density Lipoprotein Cholesterol; DBP: Diastolic Blood Pressure; Daily Energy Intake; rMED: Relative Mediterranean Diet; RRR: Relative Risk Ratios.
